# The effect of curcumin (turmeric) on Alzheimer's disease: An overview

**DOI:** 10.4103/0972-2327.40220

**Published:** 2008

**Authors:** Shrikant Mishra, Kalpana Palanivelu

**Affiliations:** Department of Neurology, VA/USC 16111, Sepulveda, CA, USA

**Keywords:** Alternative approach to Alzheimer's, beta amyloid plaques, curcumin, curcumin and dementia, epidemiology, turmeric

## Abstract

This paper discusses the effects of curcumin on patients with Alzheimer's disease (AD). Curcumin (Turmeric), an ancient Indian herb used in curry powder, has been extensively studied in modern medicine and Indian systems of medicine for the treatment of various medical conditions, including cystic fibrosis, haemorrhoids, gastric ulcer, colon cancer, breast cancer, atherosclerosis, liver diseases and arthritis. It has been used in various types of treatments for dementia and traumatic brain injury. Curcumin also has a potential role in the prevention and treatment of AD. Curcumin as an antioxidant, anti-inflammatory and lipophilic action improves the cognitive functions in patients with AD. A growing body of evidence indicates that oxidative stress, free radicals, beta amyloid, cerebral deregulation caused by bio-metal toxicity and abnormal inflammatory reactions contribute to the key event in Alzheimer's disease pathology. Due to various effects of curcumin, such as decreased Beta-amyloid plaques, delayed degradation of neurons, metal-chelation, anti-inflammatory, antioxidant and decreased microglia formation, the overall memory in patients with AD has improved. This paper reviews the various mechanisms of actions of curcumin in AD and pathology.

## Introduction

### Alzheimer's disease

Alzheimer's disease (AD) is a progressive neurodegenerative disease. It is characterized by progressive cognitive deterioration together with declining activities of daily living and behavioral changes. It is the most common type of pre-senile and senile dementia. According to the World Health Organization (WHO), 5% of men and 6% of woman of above the age of 60 years are affected with Alzheimer's type dementia worldwide.[1] In India, the total prevalence of dementia per 1000 people is 33.6%, of which AD constitutes approximately 54% and vascular dementia constitutes approximately 39%. AD affects approximately 4.5 million people in the United States or approximately 10% of the population over the age of 65, and this number is projected to reach four times by 2050. The frequency increases to 50% by the age of 80 years. Every year more than $100 billion is spent for health care in the U.S. to treat AD in primary care settings alone.

#### Neuropathology of AD:

**Figure 1:**
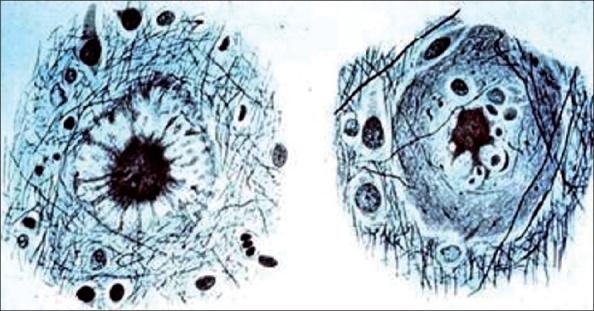
Neuritic plaques are one of the characteristic structural abnormalities found in the brains of Alzheimer patients

The neuropathological process consists of neuronal loss and atrophy, principally in the temporoparietal and frontal cortex, with an inflammatory response to the deposition of amyloid plaques and an abnormal cluster of protein fragments and tangled bundles of fibres (neurofibillary tangles). Neurotic plaques are relatively insoluble dense cores of 5-10 nm thick amyloid fibrils with a pallor staining “halo” surrounded by dystrophic neuritis, reactive astrocytes and activated microglia. There is an increased presence of monocytes/macrophages in the cerebral vessel wall and reactive or activated microglial cells in the adjacent parenchyma.[2,3] The main protein component of amyloid in AD is the 39-42 amino acid (beta) amyloid peptide (A-beta) [Figure 1].

### Curcumin

**Figure 2:**
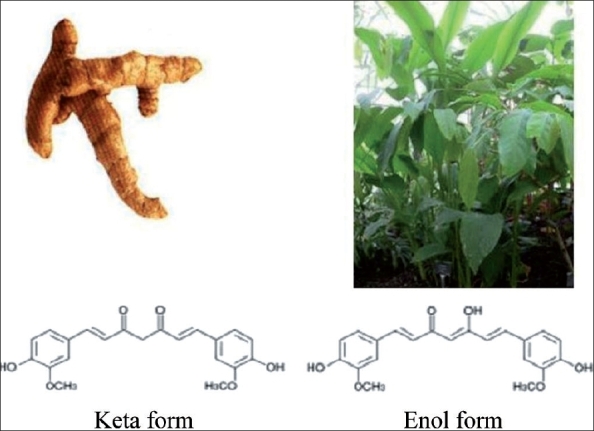
(2a) Turmeric, (2b) Turmeric plant, (2c) Keto and enol form of curcumin

Curcumin (Curcuma longa - Haldi) is the source of the spice Turmeric [Figure 2] and is used in curries and other spicy dishes from India, Asia and the Middle East. Similar to many other herbal remedies, people first used curcumin as a food and later discovered that it also had impressive medicinal qualities. It has been used extensively in Ayurveda (Indian system of Medicine) for centuries as a pain relieving, anti-inflammatory agent to relieve pain and inflammation in the skin and muscles. It has also proven to have anti-cancer properties.[4,5] Curcumin holds a high place in Ayurvedic medicine as a “cleanser of the body,” and today, science is finding a growing list of diseased conditions that can be healed by the active ingredients of turmeric.[6]

## The Plant

Botanical name: Curcuma longa; Family: Zingiberaceae, the ginger family. Turmeric is a sterile plant and does not produce any seeds [Figure 2]. The plant grows up to 3-5 ft tall and has dull yellow flowers. The underground rhizomes or roots of the plant are used for medicinal and food preparation. The rhizome is an underground stem that is thick and fleshy ringed with the bases of old leaves. Rhizomes are boiled and then dried and ground to make the distinctive bright yellow spice, turmeric.

### 

#### Turmeric History:

Probably originating from India, turmeric has been used in India for at least 2500 years. It is most common in southern Asia and particularly in India. Turmeric was probably cultivated at first as a dye and later on it was used as cosmetic and as an auspicious and aromatic food substance. It possesses antiseptic, anti-inflammatory detoxifying properties as well as carminative properties. Turmeric has a long history of medicinal use in South Asia and was widely used in Ayurvedic, Siddha and Unani systems. It is thought to be a hybrid selection and vegetative propagation of wild turmeric (Curcuma aromatica), which is native to India, Sri Lanka and the eastern Himalayas and some other closely related species.

## Curcumin and Alzheimer's Disease

Worldwide, there are over 1000 published animal and human studies, both *in vivo* and *in vitro* in which the effects of curcumin on various diseases have been examined. Studies include epidemiological, basic and clinical research on AD.

**Table:** Bio Chemical properties

**Property name**	**Property value**	**Notes/description**
Molecular formula	C_21_ H_20_ O_6_	Molecular formula for curcumin
Main curcuminoids present in turmeric	Curcumin Demethoxycurcumin Bisdemethoxycurcumin	Curcumin is the main curcuminoid and it is considered to be most active constituent.
		The curcuminoid are polyphenols and are responsible for the yellow color of turmeric.
Tautomeric forms of curcumin	keto and enol[7] [Figure 2–3] It can also exist as *cis-trans* isomeric forms.[8]	The enol form is more energetically stable in the solid phase and in solution. Curcumin can be used for boron quantification in the so-called curcumin method. It reacts with boric acid forming a red color compound known as rosocyanine. It has photo-biological and photosensitizing activity.
E number	E100	As curcumin is brightly colored, it may be used as a food color. It is often used as food additive.
Melting point	183°C (361 K)	
Color	It can appears as bright yellow to orange powder	
Molar mass	368.38 g/mol	
Systemic name	(*1E,6E*)-1,7-bis(4-hydroxy-3-methoxyphenyl)-1,6-heptadiene-3,5-dione)	Other names for the curcumin are curcumin diferuloylmethane, C.I. 75300, Natural Yellow 3

## Epidemiological Studies

Various studies and research[9,10] results indicate a lower incidence and prevalence of AD in India. The prevalence of AD among adults aged 70-79 years in India is 4.4 times less than that of adults aged 70-79 years in the United States.[9] Researchers investigated the association between the curry consumption and cognitive level in 1010 Asians between 60 and 93 years of age. The study found that those who occasionally ate curry (less than once a month) and often (more than once a month) performed better on a standard test (MMSE) of cognitive function than those who ate curry never or rarely.[10]

### 

#### Mechanism of action of curcumin on Alzheimer's disease:

**Figure 3:**
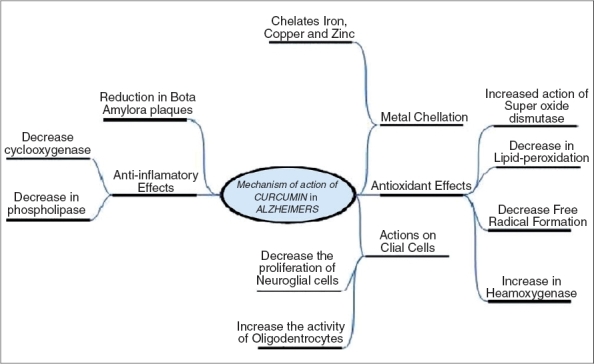
Different mechanisms of action of curcumin in AD

The process through which AD degrades the nerve cells is believed to involve certain properties: inflammation, oxidative damage and most notably, the formation of beta-amyloid plaques, metal toxicity [Figure 3]. There have been several studies on effects of curcumin on AD. Outlined below are some of the studies and their conclusions.

## Effects of Curcumin on Macrophages

A study conducted at UCLA found that curcumin may help the macrophages to clear the amyloid plaques found in Alzheimer's disease. Macrophages play an important role in the immune system. They help the body to fight against foreign proteins and then effectively clear them. Curcumin was treated with macrophages in blood taken from nine volunteers: six AD patients and three healthy controls. Beta amyloid was then introduced. The AD patients, whose macrophages were treated with curcumin, when compared with patients whose macrophages were not treated with curcumin, showed an improved uptake and ingestion of the plaques. Thus, curcumin may support the immune system to clear the amyloid protein.[11]

### 

#### Curcumin on glial cells:

Recent histological studies reveal the presence of activated microglia and reactive astrocytes around A-beta plaques in brains from patients with AD. The chronic activation of microglia secretes cytokines and some reactive substances that exacerbate A-beta pathology. So neuroglia is an important part in the pathogenesis of AD. Curcumin has a lipophilic property and can pass through all cell membranes and thus exerts its intracellular effects. Curcumin has anti-proliferative actions on microglia. A minimal dose of curcumin affects neuroglial proliferation and differentiation. Its inhibition of microglial proliferation and differentiation were studied and researched by the University of Southern California Los Angeles (UCLA). Researchers[12] using doses of 4, 5, 10, 15, 20 microM concentration of curcumin in C-6 rat glioma 2B-clone cells, a mixed colony of both neuroglial cells in a six- day trial, showed that curcumin dose dependently stops the proliferation of neuroglial cells, by differentiate into a mature cell or undergo apoptosis. It inhibits neuroglial cells proliferation dose dependently (i.e.) higher the concentration, the greater the inhibition. It has shown to decrease the glutamine synthetase (GS) assay, a marker enzyme for astrocytes. In the same study, curcumin was shown to increase CNP (2′3′- cyclic Nucleotide 3′-phosphohydrolase), a marker enzyme for oligodendrocytes. The overall effect of curcumin on neuroglial cells involves decreased astrocytes proliferation, improved myelogenesis and increased activity and differentiation of oligodendrocytes.

## Curcumin as an Anti Inflammatory in Alzheimer's

One of the important pathogenesis in Alzheimer's disease is the chronic inflammation of nerve cells. Several studies have demonstrated the associated inflammatory changes such as microgliosis, astrocytosis and the presence of pro-inflammatory substances that accompany the deposition of amyloid-β (Aβ) peptide. Patients with the prolonged use of certain nonsteroidal anti-inflammatory (NSAID) drugs such as ibuprofen have been shown to have a reduced risk of developing the symptoms of AD; however, the chronic use of NSAID can cause a toxic effect on the kidneys, liver and GI track. Curcumin has a potent anti-inflammatory effect. Through its various anti-inflammatory effects, it may have a role in the cure of AD. Curcumin inhibits Aβ-induced expression of Egr-1 protein and Egr-1 DNA-binding activity in THP-1 monocytic cells. Studies have shown the role of Egr-1 in amyloid peptide-induced cytochemokine gene expression in monocytes. By inhibition of Egr-1 DNA-binding activity by curcumin, it reduces the inflammation. The chemotaxis of monocytes, which can occur in response to chemokines from activated microglia and astrocytes in the brain, can be decreased by curcumin.[13,14]

Curcumin is found to inhibit cyclooxygenase (COX-2), phospholipases, transcription factor and enzymes involved in metabolizing the membrane phospholipids into prostaglandins. The reduction of the release of ROS by stimulated neutrophils, inhibition of AP-1 and NF-Kappa B inhibit the activation of the pro-inflammatory cytokines TNF (tumor necrosis factor)-alpha and IL (interleukin)-1 beta.[15,16] Overall, curcumin decreases the main chemical for inflammation and the transcription of inflammatory cytokines. Curcumin inhibits intracellular IL-12 p40/p70 and IL-12 p70 expression. The exposure to curcumin also impaired the production of pro-inflammatory cytokines (IL-1, IL-6 and TNF-). These studies indicate a potent inhibitor of pro-inflammatory cytokine production by curcumin and it may differ according to the nature of the target cells.

## Curcumin as an Anti-oxidant

Curcumin inhibits the activity of AP-1, a transcription factor involved in expression of amyloid, which is linked to AD. Curcuminoids are proven to have strong antioxidant action demonstrated by the inhibition of the formation and propagation of free radicals. It decreases the low-density lipoprotein oxidation and the free radicals that cause the deterioration of neurons, not only in AD but also in other neuron degenerative disorders such as Huntington's and Parkinson's disease.[16] In one study, curcuma oil (500 mg Kg(-1) i.p.) was given 15 min before 2 h middle cerebral artery occlusion, followed by 24 h reflow in rats. This significantly diminished the infarct volume, improved neurological deficit and counteracted oxidative stress.[17]

A study conducted at Nanjing Medical University (China) showed that a single injection of curcumin (1 and 2 mg/kg, i.v.) after focal cerebral ischemia/reperfusion in rats significantly diminished the infarct volume, improved neurological deficit, decreased mortality and reduced the water content in the brain.[18]

Curcumin has powerful antioxidant and anti-inflammatory properties; according to the scientists, these properties believe help ease Alzheimer's symptoms caused by oxidation and inflammation.[19] A study conducted at Jawaharlal Nehru University (India) demonstrated that the administration of curcumin significantly reduced lipid peroxidation and lipofuscin accumulation that is normally increased with aging.[20] It also increased the activity of superoxide dismutase, sodium-potassium ATPase that normally decreased with aging. In another study, curcumin has been shown to protect the cells from betaA (1-42) insult through antioxidant pathway.[21] Curcumin protects brain mitochondria against various oxidative stress. Pre-treatment with curcumin protects brain mitochondria against peroxynitrite (a product of the reaction of nitric oxide with superoxide) a potent and versatile oxidant that can attack a wide range of cells *in vitro* by direct detoxification and *in vivo* by the elevation of total cellular glutathione levels.[22]

## Curcumin on Haemoxygenase Pathway

Natural antioxidant curcumin has been identified as a potent inducer of hemoxygenase, a protein that provides efficient cytoprotection against various forms of oxidative stress. By promoting the inactivation of Nrf2-keap1 complex and increased binding to no-1ARE, curcumin induces hemoxygenase activity. The incubation of astrocytes with curcumin at a concentration that promoted hemoxygenase activity resulted in an early increase in reduced glutathione, followed by a significant elevation in oxidized glutathione content.[23–25] Glutathione is an important water-phase antioxidant and essential cofactor for antioxidant enzymes protecting the mitochondria against endogenous oxygen radicals. Its level reflects the free radical scavenging capacity of the body. GSH depletion leads to tissue damage due to lipid peroxidation and oxidative damage.

## Beta-Amyloid Plaques

The most prominent characteristic feature in AD is the presence of beta-amyloid plaques. These plaques are basically an accumulation of small fibers called beta amyloid fibrils. Because the deposition of beta-amyloid protein is a consistent pathological hallmark of brains affected by AD, the inhibition of A-beta generation, prevention of A-beta fibril formation, destabilization of pre-formed A-beta would be an attractive therapeutic strategy for the treatment of AD. The levels of beta-amyloid in AD mice that were given low doses of curcumin were decreased by around 40% in comparison to those that were not treated with curcumin. In addition, low doses of curcumin also caused a 43% decrease in the so-called “plaque burden” that these beta-amyloid have on the brains of AD mice. Surprisingly low doses of curcumin given over longer period were actually more effective than high doses in combating the neurodegenerative process of AD.[26] At higher concentration, curcumin binds to amyloid beta and blocks its self assembly. The key chemical features in amyloid beta are the presence of two aromatic end groups and any alterations in these groups has profound effect on its activity.

Because of the lipophilic nature of curcumin, it crosses the blood brain barrier and binds to plaques. Curcumin was a better A-beta 40 aggregation inhibitor and it destabilizes the A-beta polymer. In *in vitro* studies, curcumin inhibits aggregation as well as disaggregates to form fibrillar A-beta 40. A Japanese study showed that using fluorescence spectroscopic analysis with thioflavin T and electron microscopic studies, curcumin destabilizes the fA-beta(1-40) and fA-beta(1-42) as well as their extension.[27] Curcumin-derived isoxazoles and pyrazoles bind to the amyloid beta peptide (Abeta) and inhibit amyloid precursor protein (APP) metabolism.[28] Curcumin given to APPswe/PS1dE9 mice for 7 days crosses the blood-brain barrier as demonstrated by muliti-photon microscopy and reduces the existing senile plaques.[29] In another study, curcumin has been shown to increase the phagocytosis of amyloid-beta, effectively clearing them from the brains of patients with AD.[30]

## Metal Chelation

Studies showed that metals can induce A-beta aggregation and toxicity and are concentrated on Alzheimer's brain. Chelators' desferroxamine and cliquinol have exhibited anti-Alzheimer's effects. A study at Capital University Beijing demonstrated the toxicity of copper on neurons. A greater amount of H_2_O_2_ was released when copper (2)-A(beta)-40 complexes were added to the xanthene oxidase system. Copper was bound to A(beta)1-40 and was observed by electron paramagnetic resonance spectroscopy. In addition, copper chelators could cause a structural transition of A(beta). There was an increase on beta sheet as well as alpha-helix when copper was introduced.[31] Another study reveals that copper and zinc bind A-beta inducing aggregation and give rise to reactive oxygen species. There was a conformational change from beta sheet to alpha helix followed by peptide oligomerization and membrane penetration, when copper (or) zinc is added to A-beta in a negatively charged lipid environment.[32] Brain iron deregulation and its association with amyloid precursor protein plaque formation are implicated in the pathology of AD.[33]

Curcumin, by interaction with heavy metals such as cadmium and lead, prevents neurotoxicity caused by these metals. The intraperitoneal injection of lead acetate in rats in the presence of curcumin was studied microscopically. The results show lead-induced damage to neurons was significantly reduced in rats injected with curcumin.[34] A study at Chinese University of Hong Kong showed that by using spectrophotometry, the curcumin effectively binds to copper, zinc and iron. In addition, curcumin binds more effectively with redox-active metals such as iron and copper than the redox-inactive zinc. It is suggested that curcumin suppresses inflammatory damage by preventing metal induction of NF-kappa.[35,36]

## Cholesterol Lowering Effect

High-fat diets and increased blood cholesterol are linked to increased amyloid plaques by the intracellular accumulation of cholestryl esters.[37] Researchers believe that by inhibiting cholesterol formation and decreasing serum peroxides, curcumin might exert beneficial effects on AD.[38]

### Safety

#### Oral bioavailability:

Curcumin has poor bioavailability. Because curcumin readily conjugated in the intestine and liver to form curcumin glucuronides.[39] In a clinical trial conducted in Taiwan, serum curcumin concentrations peaked one to two hours after an oral dose. Peak serum concentrations were 0.5, 0.6 and 1.8 micromoles/L at doses of 4, 6 and 8 g/day respectively.[40] It is also measured in urine at a dose of 3.6 g/day. Absorption is poor following ingestion in mice and rats. 38% to 75% of an ingested dose of curcumin is excreted in the feces. Absorption appears to be better with food. Curcumin crosses the blood brain barrier and is detected in CSF.

### Side Effect

No apparent side effects have been reported thus far. GI upset, chest tightness, skin rashes, swollen skin are said to occur with high dose. A few cases of allergic contact dermatitis from curcumin have been reported.[41]

The chronic use of curcumin can cause liver toxicity. For this reason, turmeric products should probably be avoided by individuals with liver disease, heavy drinkers and those who take prescription medications that are metabolized by liver. Curcumin was found to be pharmacologically safe in human clinical trials with doses up to 10 g/day. A phase 1 human trial with 25 subjects using up to 8000 mg of curcumin per day for three months found no toxicity from curcumin.[42]

### Interaction

Curcumin is said to interact with certain drugs such as blood thinning agents, NSAIDs, reserpin. Co-supplementation with 20 mg of piperine (extracted from black pepper) significantly increase the bioavailablity of curcumin by 2000%.[43]

### Contraindication

Curcumin is not recommended for persons with biliary tract obstruction because it stimulates bile secretion. It is also not recommended for people with gallstones, obstructive jaundice and acute biliary colic. Curcumin supplementation of 20-40 mg have been reported to increase gallbladder contractions in healthy people.[44,45]

## Human

Epidemiological studies have shown that prevalence of AD is 4.4 lower amongst Indian Asians as compared to people of western origin.[9] *D* ementia incidence in western countries (*P* < 0.21) and East Asian countries were lower than that of Europe (*P* < 0.0004).[49]

**Table:** Experimental studies: Statistical significance

**Subject studies**	***Vitro***	***Vivo***
Mice	Curcumin was a A-beta40 aggregation inhibitor prevented A-beta42 oligomer formation and toxicity between 0.1-1.0 microM.	Given to aged Tg2576 mice with advanced amyloid accumulation, curcumin reduced amyloid levels and plaque burden reduction in plaque burden (*p* > 0.0001)[46]
	These changes were statistically significant *p* value (*p* > 0.001)[26]	
Rat	The middle-aged rats (n = 10) that were fed with various diets were evaluated for spatial memory deficits in a standard Morris water maze, showed that A_infused rats fed with curcumin (500 ppm) showed reduced path length and latency in finding the hidden platform and increase in spatial memory. (*p* < 0.001). The synotopsin loss was signi. cantly reduced in A-infused rats fed curcumin (*p* < 0.05) and Curcumin and ibuprofen reduced microglial area in both cortical layers (*p* < 0.05)	(1) Rats injected intraperitoneally with lead acetate (20 mg/kg) in the presence and absence of curcumin (30 mg/kg) were compared. Lead-induced damage to neurons signi. cantly reduced (*p* < 0.001) when studied microscopically to determine the extent of lead-induced damage to the cells in the hippocampus.[34] (2) 500 ppm of Curcumin in the diet for 4 weeks reduced the oxidative damage in rats (n = 8/group) with mild fluid percussion brain injury. Curcumin supplementation counteracted the impairment in cognition caused by traumatic brain injury with (*p* < 0.05).[47]
Planaria	Curcumin improved the memory curves in planaria with correlation coef. cient of 0.97.[48]	

*Clinical -Vivo:* Blood from six patients with AD and three healthy controls was taken and the macrophage cells were isolated. After treatment of macrophages with curcuminoids, Aβ uptake by macrophages of three of the six AD patients was found to have significantly increased (*P* < 0.001 to 0.081).[11]

Five animal and two human studies showed statistically significant *P* values.

## Conclusion

Based on the main findings detailed above, curcumin will lead to a promising treatment for Alzheimer's disease. The clinically studied chemical properties of curcumin and its various effects on AD shows the possibility to do further research and develop better drugs based on curcumin for treating AD. The recent review paper of John Ringman also supports some of the abovementioned properties of curcumin in AD;[50] however, large-scale human studies are required to identify the prophylactic and therapeutic effect of curcumin.

Several unanswered questions remain: What is the one main chemical property of curcumin that can be exploited in treating AD? What is the role of curcumin in other neurological disorders such as Parkinson's, Huntington's and other dementias? How does curcumin interact with neuronal plaques? Is it effective only as a food additive? Would it be effective when used alone or with other anti inflammatory drugs?
